# Recruitment Potential of a Green Alga *Ulva flexuosa* Wulfen Dark Preserved Zoospore and Its Development

**DOI:** 10.1371/journal.pone.0032651

**Published:** 2012-03-05

**Authors:** Temjensangba Imchen

**Affiliations:** National Institute of Oceanography (Council of Scientific and Industrial Research), Goa, India; University of Melbourne, Australia

## Abstract

The recruitment potential and the ability of *Ulva flexuosa* Wulfen zoospores to survive darkness were tested under different conditions in the present study. The dark preserved zoospore was cultured under a two-factor experimental design to test the effect of salinity and nitrate, effect of salinity and phosphate, effect of light and salinity, and effect of light and phosphate. The recruitment (germination and growth) of zoospores was significantly affected by light and salinity. The nitrate concentration of 20 µmol.l^−1^ was found to initiate the process of germination and its subsequent growth and, its effect appeared greatest under 25 psu condition. While nitrate enhances the growth of biomass more than phosphate, both show a positive interactive effect on biomass increase when crossed with salinity. The combined effect of 25 psu salinity and 8 µmol.l^−1^ phosphate exhibited higher biomass growth. There was a significant effect of light and salinity on the biomass of zoospore, though there was no significant interaction between the two factors. There was an increase in biomass of growing zoospores to increase in light intensity and 80 µmol.m^−2^.s^−1^ of light intensity was considered optimal. Similarly, high light intensity condition favored higher biomass growth and there was significant interaction between light (80 µmol. m^−2^. s^−1^) and phosphate (4 µmol. l^−1^) in high salinity (35 psu) condition. The result of this study showed that dark preserved zoospores of *U. flexuosa* have the potential for recruitment and it gives us an understanding how different factors play a role in the process of recruitment.

## Introduction

*Ulva flexuosa* Wulfen (syn. *Enteromorpha flexuosa* (Wulfen) J. Agardh) is an annual filamentous green alga, considered as an opportunistic species and has a highly successful reproductive rate. The reproductive cells are known to have an ability to photosynthesize and release propagules daily under favorable conditions. This seaweed is widely distributed due to its wide range of tolerance to salinity and water temperature [1], [2]. Beside this, they are implicated as an invasive and fouling species [3]. A congeneric species of *Enteromorpha* was found to cause a huge bloom in China recently [2], [4].

The spores as dispersal agents of *Enteromorpha* sp. can be found in the water column of coastal water in large quantities [5] and they are known to survive up to 8 days depending on the prevailing environmental condition [6]. From the experimental results [5], [7], [8], [9], [10] by an earlier worker, the propagules and spores of *Enteromorpha* sp. have been reported to survive in the dark and in a reduced light condition for many days. However, the ability of zoospores to survive in dark condition appears unrelated to taxonomic groups, life history or propagule size [9]. The ability of zoospores to survive darkness serves as a spore/propagule bank for future recruitment. The possibility of this condition may occur due to the intake of ballast water from the coastal region which may serve as a spore/propagule bank during the transit period. Several annual species of macroalgae have the potential to cause a massive growth called bloom in eutrophic coastal waters causing great damage to the native habitat building vegetation and its associated component [11], [12], [13].

Recruitment and growth of ephemeral macroalgae are influenced by multiple environmental factors like light, temperature and salinity [13], [14]. The recruitment potential of dark preserved *Ulva flexuosa* zoospores, its ability to germinate and grow under different salinity, light and nutrient conditions of laboratory set up was investigated in the present study.

## Materials and Methods

The mature thalli of *U. flexuosa* were collected from the intertidal rocky shores of Vagator, Goa, India (15°35′53″N 73°44′41″E). The zoospores were obtained by the dehydration method as described [15]. The algal material was thoroughly washed with sterile seawater, which was gently squeezed to remove excess water and blotted dry. It was kept in a dry petri dish under fluorescent white light (50 µmol m^−2^ s^−1^) at room temperature (24±1°C). The algal sample was then covered with filtered seawater after twelve hours to facilitate the release of typically a dense suspension of zoospores. The zoospore suspension was then transferred to a cryo vial (1.5 ml) wrapped in dark polythene and stored at 4°C. The dark preserved zoospores were used at the end of 10 months. An experimental design of two-factor as described by [16] (Table 1) was performed under laboratory condition to test the recruitment potential of the dark preserved zoospores of *U. flexuosa*. The effect of nutrient on the growth of zoospore was checked using filtered sea water (0.22 µM) collected from Vagator beach (analysis showed to contain <0.5 µM m^−3^ NO_3_^−^, <1.8 µM m^−3^ PO_4_^−^). The culture medium of modified Provasoli medium [17] was prepared from this water with no added Nitrate or Phosphate source. The medium was enriched with Nitrate and Phosphate using a stock solution of Sodium Nitrate (30,000 µmol.l^−1^) and Sodium diHydrogen Phosphate (7500 µmol.l^−1^). To study the effect of nutrient, three concentrations of nitrate were 20, 80 and 150 µmol.l^−1^, while it was 4, 8 and 15 µmol l^−1^ respectively for phosphate. Germanium dioxide (0.5 mg.l^−1^) was added to the medium to control the growth of diatoms [14]. Three different levels of salinity (15, 25 and 35 psu) were used to study the effect of salinity and it was adjusted by distilled water. The zoospore suspension (0.5 ml. l^−1^) was then added to the culture vials containing a medium of 10 ml. The culture medium was renewed every fifth day. The culture was maintained for 30 days at a temperature of 23±1°C, the dark/light period of 14/10 and the light was provided by white fluorescent tubes (Philips). To study the effect of light, three different levels of light intensity were used (20, 40 and 80 µmol. m^−2^. s^−1^). The requirement of different light intensity was set up with the help of a digital Lux meter (Sigma Instruments, India), a device use for measuring light intensity. The cultures were maintained under 40 µmol.m^−2^.s^−1^ light condition, when light was not a factor.

**Table 1:** Simplified scheme of two-factorial experiment.

Sl. No	Factors	Constant factor
1	Salinity×Nitrate	Phosphate
2	Salinity×Phosphate	Nitrate
3	Light×Salinity	Nitrate & Phosphate
4	Light×Phosphate	Nitrate & Salinity

The assessment of developing zoospore biomass was done by chlorophyll a measurement. Before the start of the experiment, initial chlorophyll concentration was measured by taking 0.5 ml. l^−1^ of zoospore suspension. The chlorophyll a was measured again at the end of the experiment to measure the growth and biomass of zoospore. The germinating zoospores in the culture vials were removed by scrubbing using a cell scraper (BD, Falcon). It was filtered in GF/F microfiber filter paper (Whatman) and chlorophyll was extracted with acetone after keeping overnight at 4°C. Chlorophyll was measured in Turner laboratory flourometer (Trilogy™) and expressed as µg.cm^−3^.

The mean growth rate of the biomass in a given culture vial was calculated by the formula: W_t_: W_0_×e^μt^, Where, W_t_ is the biomass of spore at the end of the experiment, W_0_ is the initial biomass (taken from 0.5 ml.l^−1^ of spore concentrate), t is the number of days of the experiment and μ is the specific growth rate [18]. The data were a mean of four replicates and the data were square root transformed to fulfill ANOVA assumption whenever required. The two-way analysis of variance was performed to determine the interaction between factors and the significance was compared by Tukey test at 0.05 probability level. All the statistical analysis was done according to [19] and was performed using STATISTICA software version 6.

## Results

### Effect of salinity and nitrate (NO_3_^−^)

**Figure 1:**
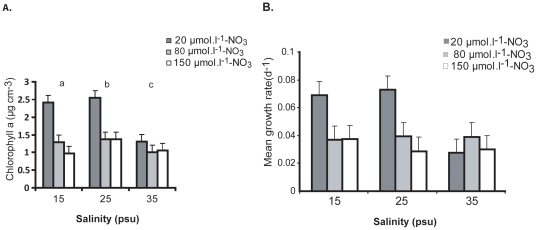
A. Measurement of Chlorophyll a concentration for the determination of growing zoospore biomass of *U. flexuosa*. Three levels of salinity and nitrate with phosphate (15 µmol^−1^) as constant. **B.** Mean growth rate (d-1) from interaction of different levels of salinities and nitrate. Bar indicates standard error ±1 (n = 4) and different letters represent significant differences between treatments.

The *U. flexuosa* growing zoospore biomass was significantly affected by salinity and nitrate (p<0. 0001). There was noteworthy interaction between salinity and nitrates (p<0. 05). Biomass of the growing zoospores increased significantly at salinity 15 psu and the highest biomass increase was at 25 psu (Fig. 1A). The highest salinity tested at 35 psu showed a decline in biomass. The growing zoospore biomass was significantly affected by the nitrate 20 µmol.l^−1^, lowest concentration used in the present study compared to two higher concentrations of 80 µmol. l^−1^ and 150 µmol. l^−1^ respectively. The optimal effect of nitrate occurred at 25 psu salinity in combination with 15 µmol. l^−1^ of phosphate (Fig. 1B).

### Effect of salinity and phosphate (PO_4_^−^)

**Figure 2:**
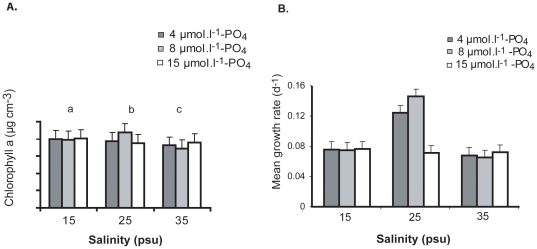
A. Measurement of Chlorophyll a concentration for the determination of growing zoospore biomass of *U. flexuosa*. Three levels of salinity and phosphate with nitrate (80 µmol^−1^) as constant. **B.** Mean growth rate (d-1) from the interaction of different levels of salinities and phosphate. Bar indicates standard error ±1 (n = 4) and different letters represent significant differences between treatments.

The biomass of growing zoospore was significantly affected (p<0.0001) by salinity and phosphate combination. The effect of phosphate concentration was similarly significant (p<0.0001). The biomass increase of growing zoospores was similar in all three different phosphate concentrations tested (Fig. 2A); however, biomass was higher at 8 µmol.l^−1^. Similarly, little difference was observed on the biomass between three levels of salinity tested. The combined effect of salinity 25 psu and 8 µmol.l^−1^ of phosphate concentration exhibited a higher biomass, thus a higher growth rate (Fig. 2B).

### Effect of light and salinity

**Figure 3:**
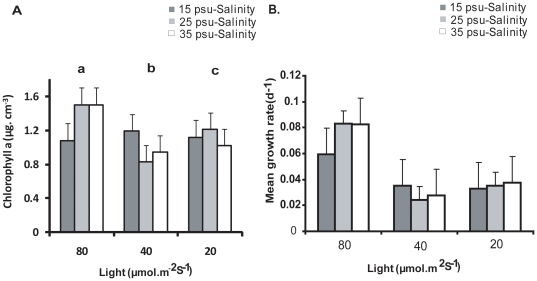
A. Measurement of Chlorophyll a concentration for the determination of growing zoospore biomass of *U. flexuosa*. Three different levels of light and salinity with both nitrate (80 µmol^−1^) and phosphate (15 µmol^−1^) as constant. **B.** Mean growth rate (d-1) from the interaction of different levels of light and salinity. Bar indicates standard error ±1 (n = 4) and different letters represent significant differences between treatments.

There was a significant increase in the biomass of growing zoospores with increase in light intensity (p<0.0001) (Fig. 3A, 3B). The biomass was highest at the highest light intensity (80 µmol. m^−2^. s^−1^) tested (p<0.0001) than the other two lower levels of light (20 and 40 µmol. m^−2^. s^−1^). Salinity also had a significant effect (p<0.0001) and salinity of 25 psu and 35 psu had considerable increase of biomass when in concert with light intensity of 80 µmol m^−2^ s^−1^, although, no significant interactions were observed between the two factors (p>0.05).

### Effect of light and phosphate (PO_4_^−^)

**Figure 4:**
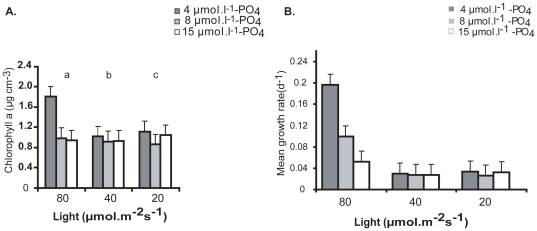
A. Measurement of Chlorophyll a concentration for the determination of growing zoospore biomass of *U. flexuosa.* Three levels of light and phosphate with nitrate (80 µmol^−1^) and salinity (35 psu) as constant. **B.** Mean growth rate (d-1) from interaction of different levels of light and phosphate. Bar indicates standard error ±1 (n = 4) and different letters represent significant differences between treatments.

There was salient interaction between light and phosphate (p<0.05). Phosphate concentrations similarly had a significant effect on the biomass of zoospores and the effect of phosphate concentration (4 µmol) was significant (p<0.0001) compared with other two concentrations in all the different levels of light. The highest biomass increase was observed at the highest irradiance condition (80 µmolm^−2^ s^−1^) in combination with a phosphate concentration (4 µmol.l^−1^) (Fig. 4A). The mean growth was similarly shown to be higher at higher light intensity compared to the other two lower light levels (Fig. 4B). However, this result is in contrast to the result from the cross between salinity and phosphate, which suggests there was an interacting effect between light and phosphate under salinity of 35 psu.

## Discussion

The influence of salinity was significant in the process of *U. flexuosa* recruitment (germination and growth). Though, *Ulva's* are known to exist in a wide range of salinity, optimal salinity for growth of adults appears to vary spatially among species [20], [21] and for spores [16]. This shows that optimum and tolerance range of salinity is largely influenced by the local condition of algal population. The salinity of 25 psu had positive effect on the growth of *Ulva* zoospores and was significantly different in all the crosses under different factors in the present study. Thus, a salinity of 25 psu was considered optimal. The other two salinity levels (15 and 35 psu) showed a varied effect but it was not significantly different from one another when crossed with phosphate. Similarly, optimum salinity was 35 psu for the increase in spore biomass of *Enteromorpha* sp. in Mondego estuary [16], while 17 and 22 psu was optimal salinity for adult growth [20]. Hypo and hypersaline condition can affect many sites in the photosynthetic machinery of algae resulting in significant changes of the photosynthetic performance [22]. On the other hand, it was observed that nitrate ameliorates the negative effect of salinity from which one could conclude that it was nutrient that govern the growth of *E. intestinalis* not salinity [23].

Favorable climatic factors like light and temperature, along with eutrophic condition promotes the proliferation and growth of the green tide forming algae like *Enteromorpha* species [24], [25], and these were believed to be the contributory factors that caused the rapid growth and proliferation of green tide forming *E. prolifera* along the coast of Qingdao [2]. Blooms are known to occur in a eutrophic condition which has a high nutrient flux; however, the results from this study show that a small concentration of nitrate in the range of 20 µmol.l^−1^ can initiate the recruitment process and its subsequent growth. The effect of nitrate was significantly different from phosphate and a biomass increase of zoospore was three times higher in nitrate feed condition. This implies that nitrate may have a potential role in regulating the growth of biomass of *U. flexuosa*. The high growth rate of the green tide forming algae was recorded in nutrient concentrations ranging from 10 to 100 µmol. l^−1^ phosphate, 100 to 1000 µmol.l^−1^ nitrate [26]. Nonetheless, both nitrate and phosphate have shown similar positive effects on biomass increase, when crossed with salinity at 25 psu. The germination and growth of zoospores increased with the increase in light intensity and the growth rate exhibited by the germinating zoospores under different levels of light regime indicates that higher irradiance level elicits higher growth response. This shows they are light sensitive and light may have a limiting effect on recruitment of zoospores at low/reduced irradiance condition. High light intensity also had a considerable effect on successful dispersal and establishment of kelp zoospores [27]. But the requirement of optimum level of light for the growth of algal germling varies from species to species [9].

The light intensities tested in the present investigation showed that high light intensity (80 µmol.m^−2^ s^−1^) positively affects the growth of zoospores. There was an increase in growth of zoospores with an increase in the availability of light. This result is in agreement with the previous work [16], [26] of light influence on *Enteromorpha* sp. recruitment, where macroalgal spore germination and growth increased with increase in light intensity. However, it was shown that there were no differences in growth of spore between 90 and 40 µmol. m^−2^. s^−1^ whenever high phosphate (6.4 µmol. l^−1^) and high salinity (35 psu) was present [16].

There was a significant interaction between light and phosphate concentration under a high salinity condition resulting in an inverse effect. When salinity 35 psu was present in high light intensity (80 µmol. m^−2^. s^−1^), low phosphate (4 µmol. l^−1^) concentration was shown to increase biomass contrary to the first experiment wherein 8 µmol. l^−1^ resulted in higher biomass. This result suggests that high salinity causes a compensatory effect to the interaction between light and phosphates resulting in low phosphate concentration to stimulate higher biomass growth which otherwise in the first experiment occurred in higher concentration. However, there was no significant difference in spore biomass in low phosphate condition even when there was a presence of high salinity [16]. While, a high nutrient load at 30 psu salinity had no detectable effect on the marine plants [28].

In conclusion, the result from the present study showed that *U. flexuosa* recruitment will be possible from the dark preserved/stored source. The study showed that zoospores of *Ulva* after a period of cold and dark requires only higher temperature, minimum of nutrients and light to cause a recruitment event. With increasing maritime traffic and less resident time between ports, this has implication for how countries and companies manage their ballast water, especially given the negative ecological effects already seen in coasts with high levels of anthropogenic disturbances, like China.
